# An arylthiazyne derivative is a potent inhibitor of lipid peroxidation and ferroptosis providing neuroprotection in vitro and in vivo

**DOI:** 10.1038/s41598-021-81741-3

**Published:** 2021-02-10

**Authors:** Meike Hedwig Keuters, Velta Keksa-Goldsteine, Hiramani Dhungana, Mikko T. Huuskonen, Yuriy Pomeshchik, Ekaterina Savchenko, Paula K. Korhonen, Yajuvinder Singh, Sara Wojciechowski, Šárka Lehtonen, Katja M. Kanninen, Tarja Malm, Jouni Sirviö, Anu Muona, Milla Koistinaho, Gundars Goldsteins, Jari Koistinaho

**Affiliations:** 1 A.I. Virtanen Institute for Molecular Sciences, University of Eastern Finland, Kuopio, Finland; 2 Aranda Pharma Ltd., Kuopio, Finland; 3 Neuroscience Center, Helsinki Institute of Life Science, University of Helsinki, P.O. Box 63, 00014 Helsinki, Finland

**Keywords:** Drug discovery, Neuroscience

## Abstract

Lipid peroxidation-initiated ferroptosis is an iron-dependent mechanism of programmed cell death taking place in neurological diseases. Here we show that a condensed benzo[b]thiazine derivative small molecule with an arylthiazine backbone (ADA-409-052) inhibits *tert*-Butyl hydroperoxide (TBHP)-induced lipid peroxidation (LP) and protects against ferroptotic cell death triggered by glutathione (GSH) depletion or glutathione peroxidase 4 (GPx4) inhibition in neuronal cell lines. In addition, ADA-409-052 suppresses pro-inflammatory activation of BV2 microglia and protects N2a neuronal cells from cell death induced by pro-inflammatory RAW 264.7 macrophages. Moreover, ADA-409-052 efficiently reduces infarct volume, edema and expression of pro-inflammatory genes in a mouse model of thromboembolic stroke. Targeting ferroptosis may be a promising therapeutic strategy in neurological diseases involving severe neuronal death and neuroinflammation.

## Introduction

Ferroptosis is an iron-dependent lipid peroxidation (LP) mechanism of non-apoptotic cell death^1^. It was recently discovered to be a major cell death pathway in neurodegenerative diseases, such as Parkinson’s disease (PD), Alzheimer’s disease (AD), and acute brain insults, including ischemic stroke and cerebral hemorrhage^2–7^. Upon injury, reactive oxygen species (ROS) and reactive nitrogen species (RNS) elevate free iron (Fe^2+^/Fe^3+^) concentration by interacting with proteins that regulate iron storage or release^8,9^. In turn, Fe^2+^/Fe^3+^ decomposes H_2_O_2_ into harmful hydroxyl radicals HO^–^ + HO^•^, thereby accelerating LP^8–10^. Fe^2+^/Fe^3+^ also blocks regeneration of glutathione (GSH), the major cellular antioxidant. GSH is essential for the activity of the enzyme glutathione peroxidase 4 (GPx4) that is exclusively responsible for the reduction of cholesterol and phospholipid hydroperoxides and esterified oxidized fatty acids^11–13^. GSH depletion and inactivation of GPx4 are key initiators of ferroptosis^14^. Importantly, in severe neurological diseases, such as stroke, ferroptosis is coupled to neuroinflammation by triggering the release of damage-associated molecular patterns (DAMPs), immunogenic lipid metabolites^15^, and pro-inflammatory cytokines from activated immune cells^3,9,16^. While the activated immune cells release multiple pro-inflammatory factors, they simultaneously generate ROS, initiating another route for LP^17,18^. Thus, seemingly interdependent pathways of redox imbalance and inflammation facilitate each other and converge into mechanisms of neuronal damage.

Recently, two ferroptosis inhibitors, ferrostatin-1 and liproxstatin-1, were shown to be protective in mouse models of transient middle cerebral artery occlusion (tMCAO)^19^ and intracerebral hemorrhage (ICH)^20^. However, these two compounds poorly cross the blood–brain barrier^2,19^ limiting their clinical development for brain diseases. Here we show that a condensed benzo[b]thiazine derivative with an arylthiazine backbone, ADA-409-052, inhibits ferroptotic cell death of neuronal cells through suppression of LP. The compound has a low molecular weight (MW: 330.33 g/mol), excellent calculated lipophilicity (Clog*P*) of 2.31 (ideal log*P* value for passive diffusion through the blood-brain barrier is 1.5–2.7^21^), and crosses well the blood-brain barrier in mice. Moreover, ADA-409-052 is strongly neuroprotective upon oral administration in a thromboembolic (TE) mouse model of stroke.

## Results

### ADA-409-052 prevents lipid peroxidation induced by TBHP treatment

**Figure 1:**
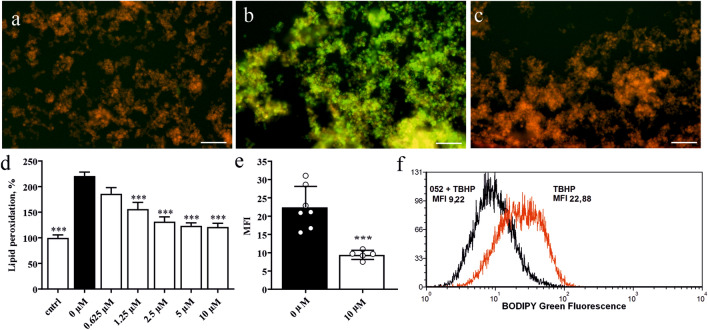
ADA-409-052 treatment protects PC-12 cells from TBHP-induced LP visualized with C11-BODIPY, a marker for cellular and intramembrane LP. **(a–c)** Representative, microscopic pictures of PC-12 cells exposed to **(a)** media control, **(b)** 1 mM TBHP, a class IV ferroptosis inducer, or **(c)** TBHP and ADA-409-052 (10 µM); scale bar: 100 µm. **(d)** Quantitative analysis of LP detected by a shift in the fluorescence signal. Data normalized to control and displayed as percentage ± s.e.m.; n = 3; One-way ANOVA with Tukey’s multiple comparison test; ****p < 0.0001. **(e)** Flow cytometry analysis of TBHP-exposed (500 µM) PC-12 cells demonstrated protection upon the addition of 10 µM ADA-409-052. Data are shown as the averaged green channel (488 nm laser, 530/30 filter) median fluorescence intensity (MFI) ± s.e.m.; n = 6/5. Unpaired, two-tailed student’s t-test; ***p < 0.001. **(f)** Representative histogram showing the median of the detected green fluorescence intensity (log values) after TBHP treatment only (red) or TBHP and ADA-409-052 (black) using flow cytometry.

PC-12 cells, exposed to 1 mM tert-Butyl hydroperoxide (TBHP), yielded massive LP, as shown by the increase of C11-BODIPY green fluorescence signal (224.3% ± 59.1% when normalized to control, p < 0.0001, Fig. 1b). In contrast, the red fluorescence signal remained predominant upon simultaneous treatment with ADA-409-052 (10 µM, Fig. 1c), which was comparable to control conditions (media only, Fig. 1a). Already the addition of ADA-409-052 at concentrations as low as of 0.625 µM suppressed some LP (to 183.7% ± 47.2%; Fig. 1d). Rising concentrations of ADA-409-052 up to 5–10 µM resulted in a potent dose-dependent reduction in TBHP-induced LP (1.25 µM: 161.12% ± 67.1%, 2.5 µM: 129.7% ± 43.5%, 5 µM: 124.5% ± 38.8%, 10 µM: 125.0% ± 49.3%; p < 0.0001; Fig. 1d). Flow cytometry analysis confirmed this reduction of TBHP-induced LP by ADA-409-052, showing a significantly lower median fluorescent signal in TBHP-exposed (500 µM) PC-12 cells after 10 µM ADA-409-052 treatment when compared with vehicle (green median fluorescent intensity (MFI) of ADA-409-052 + TBHP: 9.56 ± 1.1; MFI of TBHP only: 20.81 ± 5.3, p = 0.0006; Fig. 1e-f).

### ADA-409-052 prevents ferroptosis induced by GPx4 inhibitor RSL3

**Figure 2:**
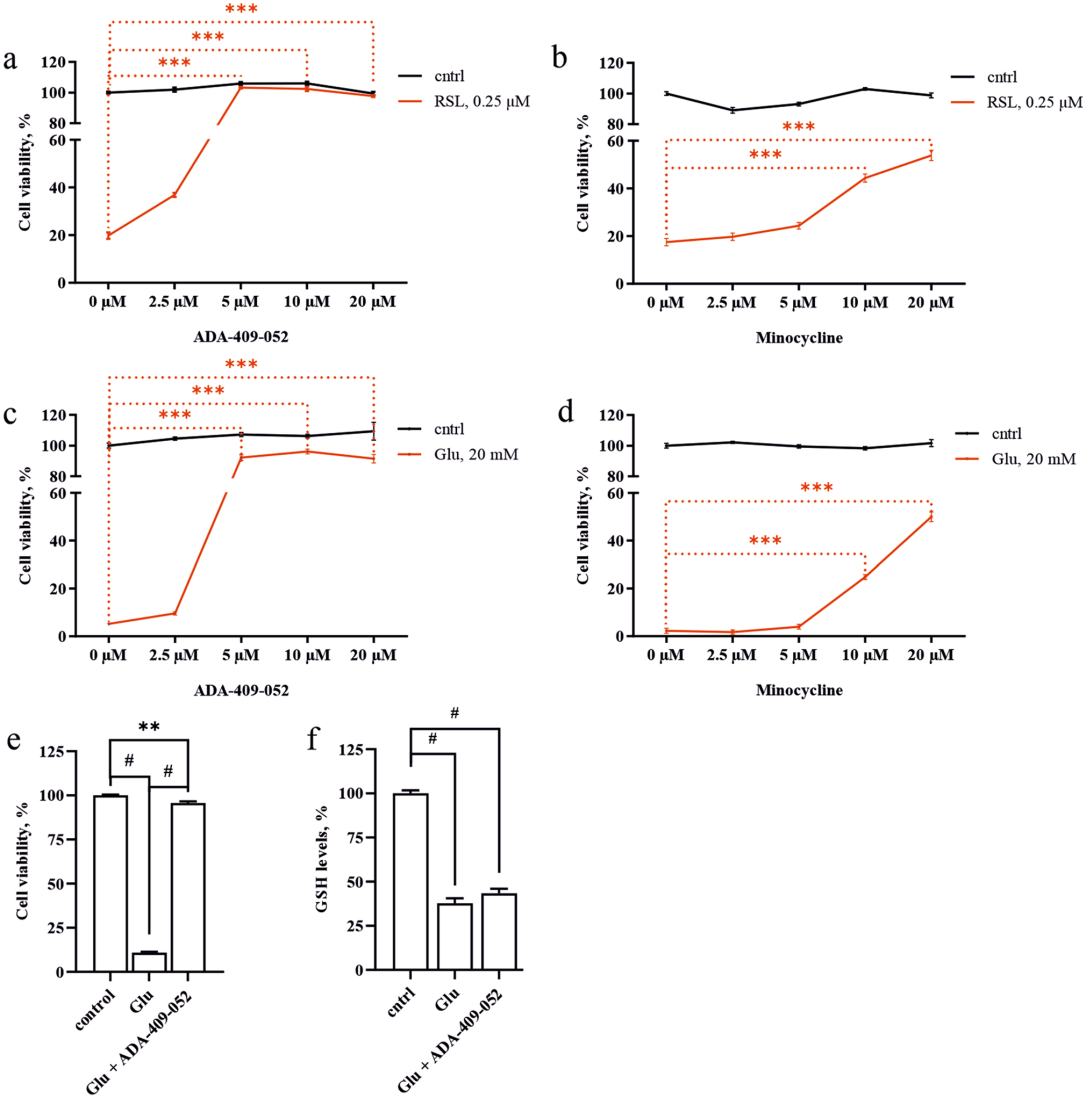
ADA-409-052 is protective against ferroptotic cell death in in vitro models of ferroptosis inducer class I and II. Increased cell viability, detected by the resazurin assay, illustrates the efficacy of ADA-409-052 on cultured PC-12 cells against either RSL3, a class II ferroptosis inducer inactivating GPx4, or glutamate, causing GSH depletion by inhibiting the system x_c_^−^. **(a)** ADA-409-052 protects PC-12 cells against RSL3-induced (0.25 µM) ferroptotic cell death significantly at all given concentrations (ADA-409-052: 2.5–20 µM). **(b)** Low concentrations of minocycline (2.5 or 5 µM) were inefficient against RSL3-mediated cell death. However, concentrations of 10 and 20 µM significantly improved cell survival. One-way ANOVA followed by Tukey’s multiple comparison test; data are presented as percentage ± s.e.m.; technical n = 6; ***p < 0.0001. **(c)** ADA-409-052 (5–20 µM) prevented glutamate-induced (20 mM) ferroptotic cell death almost entirely. **(d)** Minocycline was only protective at concentrations of 10 and 20 µM. One-way ANOVA followed by Tukey’s multiple comparison test; data are presented as percentage ± s.e.m.; technical n = 6; ***p < 0.0001. **(e,f)** Glutamate-induced reduction of intracellular GSH-levels remains unchanged by ADA-409-052. Exposure of PC-12 cells to glutamate (20 mM) caused a major reduction of cell viability **(f)** and GSH levels **(e)** when compared with untreated control cells. While the cell viability was rescued almost entirely by the addition of ADA-409-052 (5 µM), the GSH levels remained comparable to glutamate-exposed cells. One-way ANOVA followed by Tukey’s multiple comparison test; data are presented as percentage ± s.e.m.; technical n = 6; **p = 0.0013; #p < 0.0001.

Next, we tested ADA-409-052′s efficacy against RSL3-induced ferroptosis in PC-12 cell cultures. The commercially available compound RSL3, a class II ferroptosis inducer, inactivates GPx4 causing ferroptotic cell death. Figure 2a shows that 2.5 µM ADA-409-052 lowered RSL3-induced (0.25 µM) ferroptotic cell death significantly (ADA-409-052: 36.89% ± 2.21% cell viability compared to RSL3 only: 19.8% ± 3.44%; p < 0.0001). Adding ≥ 5 µM of ADA-409-052 rescued about ~ 100% of the RSL3-treated cell population (ADA-409-052: 5 µM: 103.21% ± 1.45%, 10 µM: 102.39% ± 3.64%, 20 µM: 97.70% ± 1.39%; p < 0.0001). In comparison, our neuroprotective positive control compound, minocycline, was inefficient at concentrations below 10 µM and remained weak also at higher concentrations, although reaching significance (minocycline: 10 µM: 44.36% ± 4.1% cell viability, p < 0.0001; 20 µM: 53.87% ± 5.3% cell viability; p < 0.0001; Fig. 2b).

### ADA-409-052 prevents ferroptosis induced by glutathione depletion

High extracellular concentrations of glutamate cause GSH-depletion by inhibiting the cysteine/glutamate antiporter system x_c_^−^^22^. This depletion inhibits GPx4-activity, thereby initiating ferroptosis. Figure 2c,d show that 20 mM glutamate alone resulted in over 90% death of PC-12 cells, while glutamate-exposure in combination with 5, 10, or 20 µM ADA-409-052 rescued more than 90% of the cells (cell survival ADA-409-052: 5 µM: 92.18% ± 4.45%, 10 µM: 96.2% ± 3.98%, 20 µM: 91.53% ± 2.52%; p < 0.0001; Fig. 2c). In contrast, minocycline was less protective at corresponding concentrations (cell survival minocycline: 10 µM: 24.86% ± 2.52%; 20 µM: 49.96% ± 5.02%; p < 0.0001, Fig. 2d). To demonstrate that the efficacy of ADA-409-052 is independent of endogenous GSH, we determined the intracellular GSH levels in glutamate-exposed PC-12 cells. We found that ADA-409-052 prevented glutamate-exposed cell death efficiently (20 mM Glu: 10.87% $\pm$ 0.57%; Glu + ADA-409-052: 95.67% ± 0.95%; p < 0.005; Fig. 2e). Also, the GSH levels in both glutamate only and glutamate plus ADA-409-052 co-treated cells were significantly reduced compared to unstimulated cells (GSH-levels of: 20 mM Glu: 37.8% ± 6.8%; ADA-409-052: 43.4% ± 6.3%, unstimulated control cells were normalized to 100%; p < 0.0001, Fig. 2f).

### ADA-409-052 prevents changes in mitochondrial morphology in glutamate-exposed PC-12 cells

**Figure 3:**
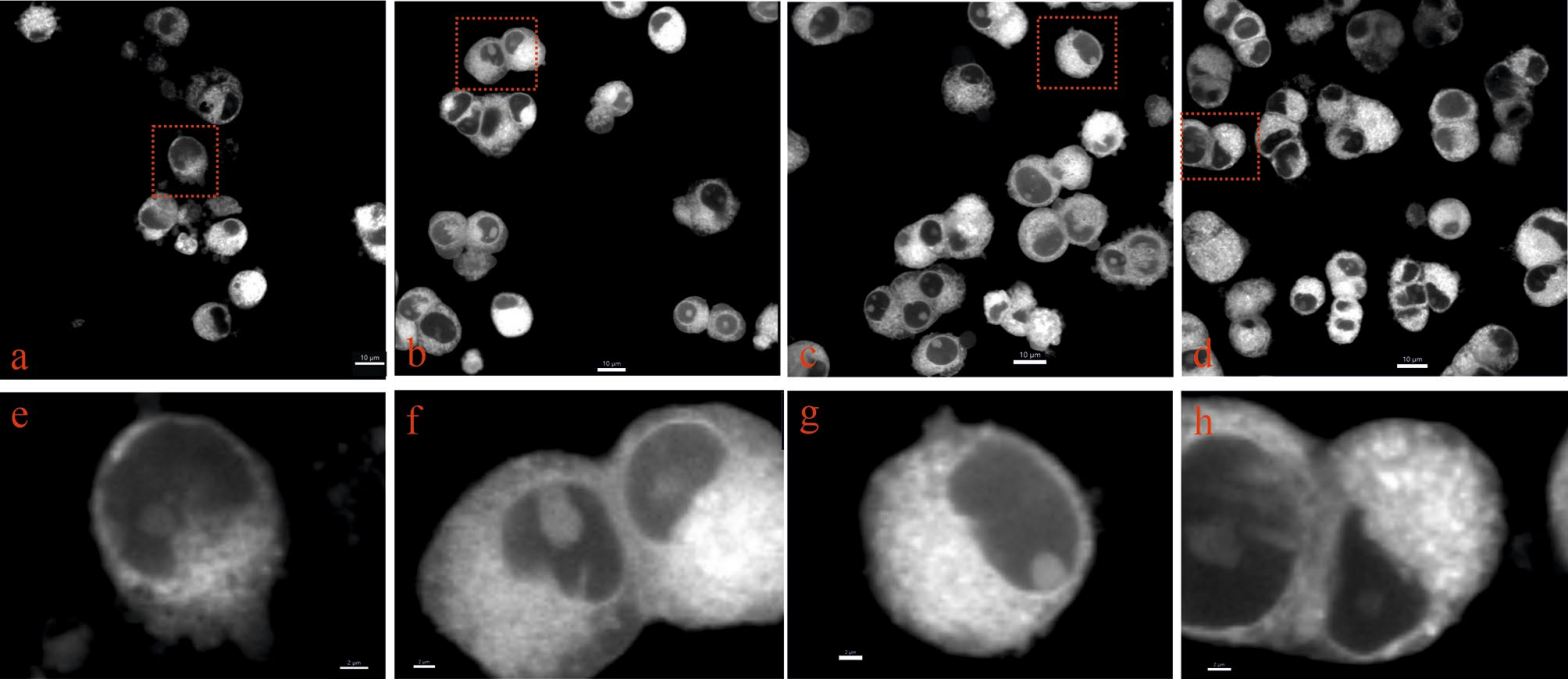
Changes in mitochondrial morphology in glutamate-exposed PC-12 cells are prevented by ADA-409-052. MitoTracker Red CMXRos was used to stain mitochondria of PC-12 cells after 24 h of exposure to **(a,e)** 20 mM glutamate, **(b,f)** glutamate and 10 µM ADA-409-502, or **(c,g)** ADA-409-052 only, plus control (**d,h**; media only). (**b–d,f–h**) Cells, exposed to ADA-409-052 in absence or presence of glutamate show a similar distribution and density of MitoTracker Red CMXRos-positive structures as control cells. (**a,e**) However, in glutamate-exposed cells appear distribution and density altered, and the overall number of viable cells reduced. Mitochondria were detected by confocal microscopy in live cells and magnified images (**e–h**) were produced using Imaris; red boxes indicate magnifications. Scale bar: 10 µm; 2 µm for magnifications.

Mitochondrial fragmentation is a major morphological alteration in ferroptosis, also when induced by high concentrations of glutamate^23–26^. We thus tested whether ADA-409-052 prevents such changes in mitochondrial morphology of PC-12 cells exposed to ferroptosis stimulation^23^. Live cell imaging of MitoTracker Red CMXRos stained mitochondria showed similar morphology of control, ADA-409-052, as well as glutamate and ADA-409-052-co-treated cells without signs of fragmentation (Fig. 3a–c,d–g). In contrast, glutamate-exposed cells show clear signs of pronounced fragmentation and reduced mitochondrial density (Fig. 3d,h), comparable with data shown elsewhere^23,26^.

### ADA-409-052 inhibits inflammation in an in vitro model of LPS stimulation

**Figure 4:**
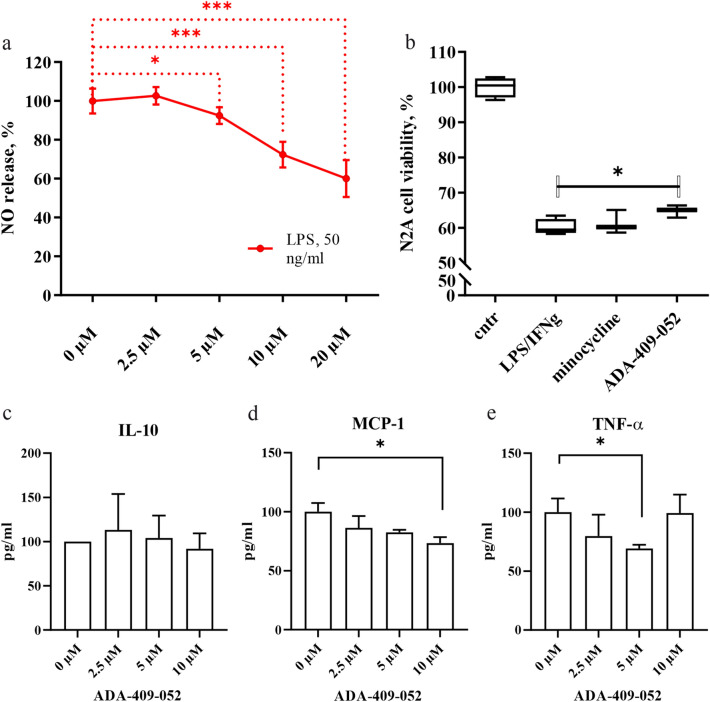
**(a)** ADA-409-052 inhibits LPS-induced inflammation in BV2 microglial-like cells. The addition of 5, 10, and 20 µM ADA-409-052 significantly attenuated the release of NO, measured from the media of LPS-exposed BV2 cells using the Griess reagent, when compared to cells treated with LPS only. Data are represented as percentages of NO-release after normalization to LPS-stimulated BV2 cells ± s.e.m.; unpaired, two-tailed student’s t-test; technical n = 6; *p < 0.05, ***p < 0.0001. (**b)** ADA-409-052 protects N2a neuronal cells when co-cultured with LPS-stimulated RAW 264.7 macrophages. Boxplots show that LPS and INF-γ treatment of co-cultured N2a cells and RAW 264.7 macrophages decreased the viability of N2a cells about 40% as detected by the CytoFLEX S (Beckman Coulter) as a fraction of CD11b negative (CD11b^-^), PI negative (PI^-^), surviving cells to the total number of gated cells, normalized to the control sample. Co-treatment of ADA-409-052 and LPS/INF-γ resulted in a significant increase of the N2a cell viability, detected by the reduced percentage of CD11b^-^/ PI positive (PI^+^, dead) cells when compared to LPS/INF-γ treatment only. In contrast, the proportion of CD11b^-^/PI^+^ N2a cells remained unaltered by minocycline. Cell survival is displayed as individual boxplot per treatment with interquartile range, whiskers set from min to max; unpaired, two-tailed student’s t-test; technical n = 3/4; *p = 0.0314. (**c–e**) ADA-409-052 alters the expression of pro- and anti-inflammatory cytokine expression of BV2 microglial cells. By measuring cytokine-secretion of ADA-409-052-stimulated BV2 cells, using the BD CBA mouse inflammation kit, **(c)** no changes in secretion of the anti-inflammatory IL-10 were detected. **(d)** However, we detected a major decrease of MCP-1 upon the addition of 10 µM ADA-409-052. **(e)** Furthermore, TNF-α decreased upon ADA-409-052-stimulation (5 µM) significantly when compared with untreated cells (0 µM ADA-409-052). Student’s t-test, n = 4; *p < 0.05.

Lipopolysaccharide (LPS) stimulates inflammatory activation of microglial BV2 cells, which is associated with a redox imbalance. We assessed the expression of inducible nitric oxide synthase (iNOS) by measuring the release of nitric oxide (NO) into the culture medium 24 h after administration of 50 ng/ml LPS. Co-administration of LPS with ADA-409-052 yielded a dose-dependent reduction in NO release (5 µM: 92.42% ± 4.38%; p = 0.0384; 10 µM: 72.33% ± 6.65%, 20 µM: 60.03% ± 9.53%; p < 0.0001; Fig. 4a).

### ADA-409-052 is neuroprotective in a co-culture model of N2a cells and stimulated RAW 264.7 macrophages

To determine whether ADA-409-052 protects against inflammation-mediated neuronal death, we exposed co-cultures of N2a cells and RAW 264.7 macrophages to 25 ng/ml LPS and 25 ng/ml interferon gamma (IFN-γ) for 24 h. The exposure resulted in about 40% neuronal cell death when compared with vehicle treatment (LPS/IFN-γ: 60.15% ± 2.28% viability; p < 0.0001). ADA-409-052 improved N2a cell viability significantly (5 µM ADA-409-052: 64.78% ± 1.77%; p = 0.0341), whereas minocycline failed to increase cell survival at the same concentration (5 µM minocycline: 61.32% ± 3.34% cell viability; p = 0.61; all Fig. 4b).

### ADA-409-052 modulates the cytokine secretion of LPS-stimulated BV2 microglial cells in vitro

By using the CBA mouse inflammation kit, we determined the effect of ADA-409-052 on the secretion of pro- and anti-inflammatory cytokines from BV2 cells. The secretion of the anti-inflammatory IL-10 remained unaltered by all used concentrations of ADA-409-052 of BV2 cells (Fig. 4c). However, the addition of 10 µM of the compound reduced the baseline (0 µM ADA-409-052) secretion of MCP-1 by BV2 cells significantly (0 µM ADA-409-052: 100% ± 7.43%, 10 µM ADA-409-052: 73.33% ± 5.14%; p = 0.026; Fig. 4d). Furthermore, the secretion of TNF-α was significantly reduced upon exposure to 5 µM when compared with control condition (5 µM ADA-409-052: 69.15% ± 3.24%, p = 0.042, Fig. 4e).

### Orally administered ADA-409-052 shows fast absorption and good brain penetration

Using a pharmacokinetic study in mice, we established an in vivo treatment protocol. Orally administered ADA-409-052 at the dose of 10 mg/kg showed individual (n = 3) maximal plasma concentrations within 0.25–1 h time window, followed by a fast distribution and plasma clearance in a time window of 2–8 h (*data not shown*). At the 0.75 h time point after orally dosing 10 mg/kg ADA-409-052, plasma and brain concentrations (n = 3) were in the ranges 1080–1324 ng/ml and 1100–1526 ng/g tissue, respectively. Oral administration of 30 mg/kg ADA-409-052 resulted in a mean plasma concentration of 3771 ng/ml and a mean brain concentration of 3237 ng/g at 0.75 h. The concentrations dropped to 23.7 ng/ml and 30.9 ng/g at 4 h, respectively, and below detection limit at 24 h (n = 3 per time point). These results indicated fast and linear dose-dependent oral exposures with excellent brain penetration with a relatively short half-life in the plasma and brain.

### Oral administration of ADA-409-052 provided protection in the mouse model of thromboembolic stroke

**Figure 5:**
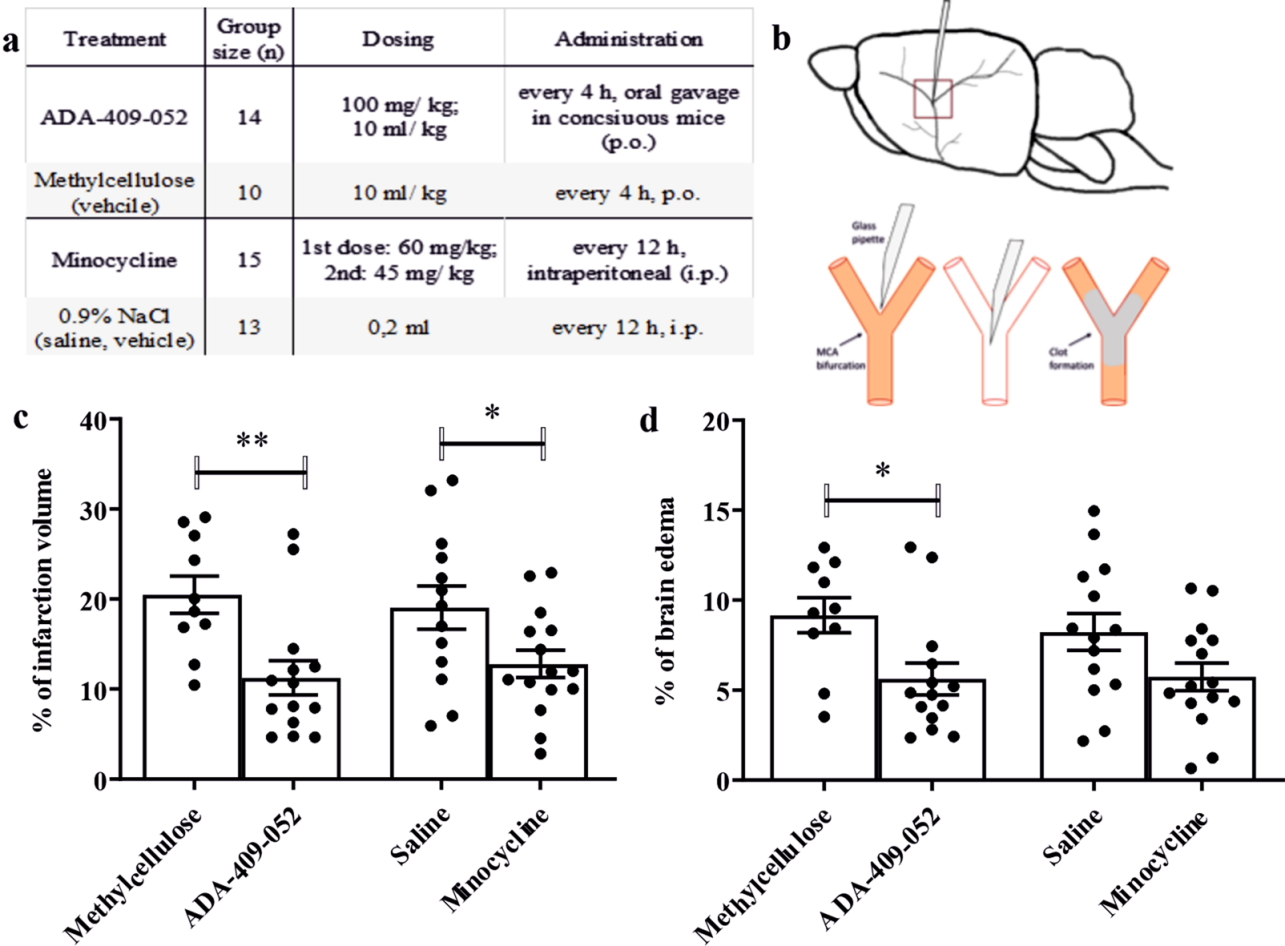
ADA-409-052 administration had a substantial neuroprotective effect in the TE-mouse model. The table **(a)** displays the group sizes, drug dosing and administration regime. ADA-409-052 was administered every 4 h, starting -1 h before TE-surgery, with 100 mg/kg at 10 ml/kg p.o. in conscious mice, methylcellulose was given accordingly. Minocycline was injected i.p. as a first dose of 60 mg/kg and a second dose of 45 mg/kg, every 12 h, whereas 0.2 ml of saline were injected i.p. to the vehicle group. Randomization was done using QuickCalcs (GraphPad Software). **(b)** shows a schematic drawing of the MCA-anatomy in the mouse brain, a magnified view below displays the inserted micropipette, and the in situ clot formation of the murine α-thrombin within the MCA (red box indicates the magnified area). **(c,d)** ADA-409-052 treatment significantly reduced infarct volume and brain edema in mice at 24 h post-ischemic stroke. Minocycline treatment, a competitor compound that was used as positive control, caused a significant reduction of the lesion volume. Of note, minocycline failed to reduce the edema size. Lesion and edema volume were assessed using T2-weighted MRI images and calculated according Shuaib’ s formula^77^. Values are presented as mean ± s.e.m.; unpaired, two-tailed student’s t-test; n = 10–15; *p < 0.05; **p < 0.005.

Next, we evaluated whether ADA-409-52 provides neuroprotection in vivo in a TE mouse model of stroke. Minocycline treatment was used as a positive control. ADA-409-052 and minocycline were repeatedly administered post-stroke (Fig. 5a,b). Mice treated with corresponding vehicles were included as negative controls. The oral dosing paradigm of ADA-409-052 was based on our pharmacokinetic study. We dosed minocycline to achieve maximal protection according previous data^27,28^. Quantification of the lesion volume by MRI analysis 24 h post-stroke demonstrated a 50% reduction in mice treated with ADA-409-052 when compared to vehicle treatment (ADA-409-052: 10.2% ± 6.1%, vehicle: 20.5% ± 6.5%; p = 0.0038; Fig. 5c). As expected, also minocycline reduced the lesion volume significantly when compared to its vehicle treatment (minocycline: 10.3% ± 4.5%, vehicle: 19% ± 8.7%; p = 0.0319; Fig. 5c). In addition and importantly, ADA-409-052 attenuated the edematous volume by 39% (ADA-409-052: 5.6% ± 3.3%, vehicle: 9.2% ± 3.1%; p = 0.0145; Fig. 5d), whereas minocycline had no significant effect on brain swelling (minocycline: 5.7% ± 3.0%, vehicle: 8.5% ± 3.9%; Fig. 5d). Blood gas levels measured from six randomly selected mice that went through the stroke operation remained unaltered by ADA-409-052 treatment, methylcellulose (vehicle) respectively (*data not shown*).

### ADA-409-052 decreased the expression of pro-inflammatory genes after ischemic stroke

**Figure 6:**
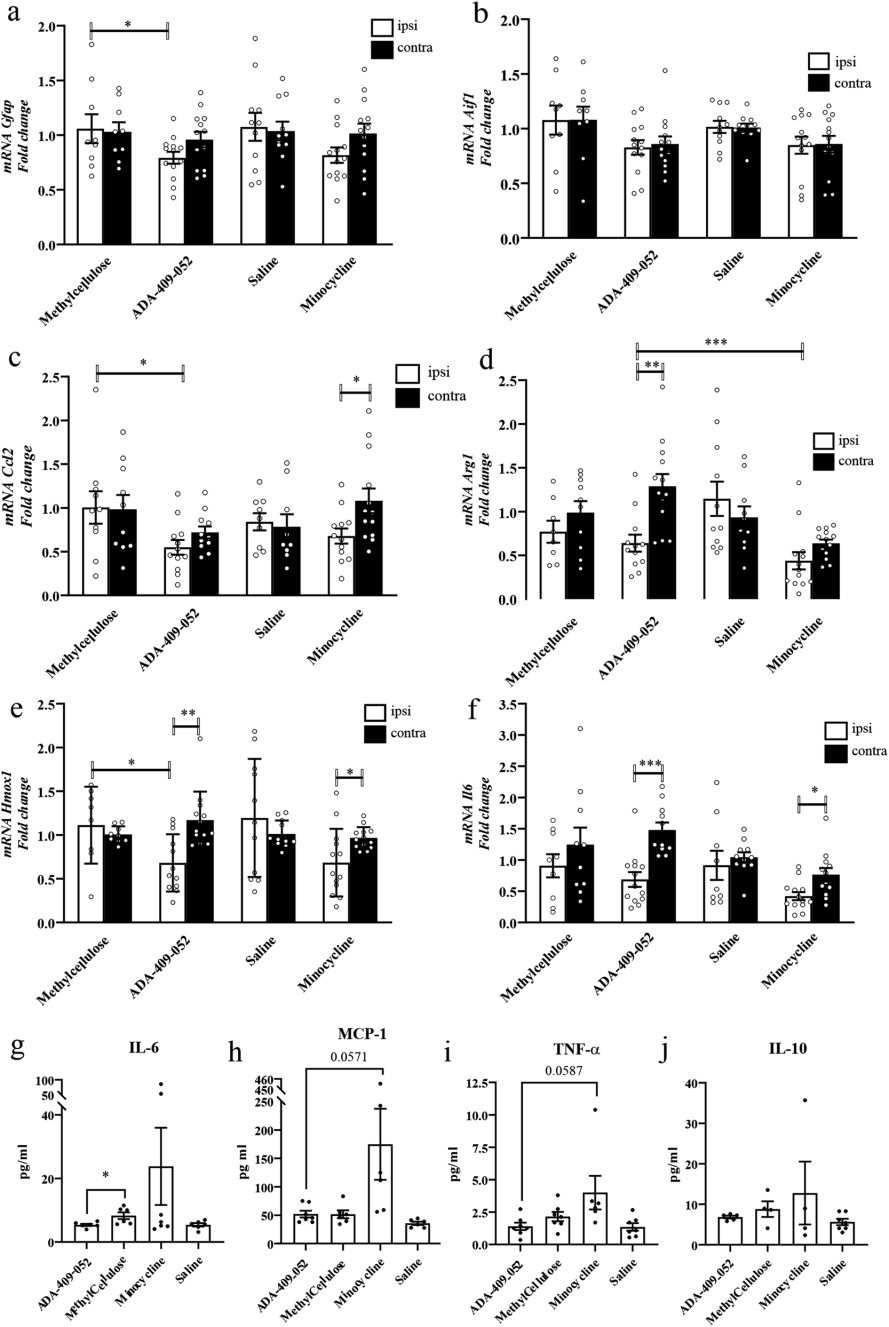
ADA-409-052 administration in vivo reduced the expression levels of pro-inflammatory markers **(a–f)** and altered the peripheral cytokine secretion **(g–j)** at 1 day post-ischemia. The mRNA expression of tissue samples from the ipsi- and contralateral peri-ischemic area at 24 h was analyzed using qPCR. Expression levels of **(a)** *Gfap* and **(c)** *Ccl2* were markedly reduced in the ipsilateral peri-ischemic area after ADA-409-052 treatment when compared to vehicle. **(b)** While, *Aif1* remained unchanged throughout all groups, ADA-409-052 lowered **(d)** *Arg1* and **(e)** *Il6* mRNA expression in the ipsilateral samples when compared to contralateral. **(f)** Ischemic mice treated with ADA-409-052 showed reduced *Hmox1* expression in the ipsilateral peri-ischemic area when compared to contralateral expression levels, to ipsilateral expression of vehicle-treated mice respectively. Minocycline decreased the expression of *Hmox1* only ipsilateral when compared to contralateral. mRNA expression levels of peri-ischemic tissue samples from ipsi- and contralateral, data are expressed as mean ± s.e.m.; unpaired, two-tailed student’s t-test; n = 9–13; *p < 0.05, **p < 0.005, ***p < 0.001. **(g)** The secretion of the inflammation-mediating IL-6 was significantly reduced in ADA-409-052-treated mice when compared to vehicle-treated mice, as detected from the blood plasma, collected from ischemic mice at 1 dpi, by using the BD CBA mouse inflammation kit. **(h,i)** Both MCP-1 and TNF-α were equally low in ADA-409-052-treated mice when compared with vehicle treatment and showed a clear trend towards reduction when compared with minocycline-treated mice (changes did not reach significance). **(j)** The peripheral secretion of IL-10 remained unaltered by any given treatment. Data are expressed as mean ± s.e.m.; unpaired, two-tailed student’s t-test; n = 7; *p = 0.027.

Stroke is well known to trigger inflammation that contributes to ischemic damage. Since ADA-409-052 provided a mild protection against inflammation-induced neuronal death in vitro, we evaluated whether ADA-409-052 ameliorates ischemia-induced inflammation by analyzing the expression of astrocytic *Gfap*, and allograft inflammatory *Aif1* encoding ionized calcium-binding adapter molecule 1 (Iba1), a commonly used microglia marker. Already during the phase of early acute inflammation at 24 h post-stroke, ADA-409-052 suppressed the stroke-induced expression of *Gfap* in the ipsilateral peri-ischemic area (from 1.059 ± 0.397 to 0.793 ± 0.197; p < 0.05; Fig. 6a), while the expression of *Aif1* remained unchanged at the given time point (Fig. 6b). However, in comparison to vehicle-treated mice, ADA-409-052 decreased the mRNA expression of *Ccl2*, in the ipsilateral perilesional tissue (ADA-409-052: 6.6 ± 0.294 to vehicle: 10.05 ± 0.584; p = 0.0278; Fig. 6c). Furthermore, the expression of *Arg1* (arginase-1) was reduced in the ipsilateral peri-ischemic area of ADA-409-052-treated mice when compared to contralateral peri-ischemic area (ipsilateral: 0.639 ± 0.334 vs contralateral: 1.288 ± 0.501; p < 0.001; Fig. 6d). Both compounds reduced the ipsilateral mRNA expression of *Il6* significantly in comparison to the contralateral expression (ADA-409-052, ipsilateral: 0.688 ± 0.425 vs contralateral: 1.479 ± 0.396; p < 0.0003; minocycline, ipsilateral: 0.422 ± 0.232 vs contralateral: 0.762 ± 0.375; p < 0.01; Fig. 6e). However, expression of *Il10* or *Tnfα* at 24 h post-stroke remained unchanged (*data not shown*). Interestingly, the expression of *Hmox1*, encoding for the enzyme heme oxygenase 1 that releases ferrous iron when catabolizing heme, was significantly reduced in the ipsilateral peri-ischemic area of ADA-409-052- and minocycline-treated mice compared with the corresponding contralateral expression (ADA-409-052, ipsilateral: 0.68 ± 0.33 vs. contralateral: 1.17 ± 0.33; p = 0.0011; minocycline, ipsilateral: 0.68 ± 0.39 vs. contralateral: 0.96 ± 0.13; p = 0.0203; Fig. 6f) and between ADA-409-052- and vehicle-treated control mice (vehicle, ipsilateral: 1.11 ± 0.44; p = 0.0212; Fig. 6f).

CBA-analyses of plasma samples, taken at 24 h post-stroke, using the mouse inflammation kit of the BD CBA, revealed significantly lower IL-6 levels in mice treated with ADA-409-052 (5.38 ± 0.354 pg/ml) when compared with vehicle-treated mice (8.27 ± 1.06 pg/ml, p = 0.027; Fig. 6g). Although the levels of MCP-1 and TNF-α appeared visibly higher in minocycline-treated mice when compared with ADA-409-052-treated mice, the averages remained statistically insignificant between all treatment groups (Fig. 6h,i). The gene expression data of *Il10*, however, are in line with the peripheral secretion into the blood plasma of the anti-inflammatory interleukin (Fig. 6j), as both remained unaltered.

## Discussion

The brain is the most susceptible mammalian tissue to the oxidative stress caused by an imbalance of redox reactions. This is due to the brain being extremely rich in lipids with unsaturated fatty acids, which are major substrates for ROS production, while simultaneously consuming about 20% of the body’s oxygen^29,30^. In addition, most brain areas have a high iron concentration, needed for iron-catalyzed processes, such as oxygen transportation, oxidative phosphorylation, myelin synthesis, and neurotransmitter metabolism^31^, leaving the brain sensitive to abnormal iron homeostasis and iron-dependent LP^32^. The risk of unbalanced iron homeostasis and iron-dependent LP is high, especially in the elderly, as iron accumulates into the brain during aging^3,33^. Moreover, abnormally high iron concentrations are found in affected brain areas in neurodegenerative diseases, such as AD, PD, amyotrophic lateral sclerosis and stroke^5,29,31,33,34^. Hence, ferroptosis is considered a major form of cell death in neurodegenerative diseases and stroke^5^, making it a potential target for therapies of age-related brain diseases.

Our study demonstrates that in neuronal cell lines ADA-409-052, a condensed benzo[b]thiazine derivative with an arylthiazine backbone, strongly inhibits LP and the subsequent cell death induced by GSH depletion or blockade of GPx4, the key-triggers of ferroptosis. Both GSH depletion and GPx4 deficiency are highly relevant conditions, as GPx4 catalyzes the reduction of lipid peroxides at the expense of GSH, and as levels of both GSH and GPx4 are reduced in stroke and neurodegenerative diseases^35^. Attempts to boost GPx4 directly or indirectly in animal models of neurodegenerative diseases and stroke have been successful^36–38^, but remained unfeasible and inefficient in clinics^39–41^. An advantage of ADA-409-052 and molecules alike is that their ability to inhibit ferroptosis is independent on the availability of GSH, suggesting that ADA-409-052 could protect the brain even when GSH levels decrease. Furthermore, ADA-409-052 inhibited glutamate-induced changes of mitochondrial morphology, facilitating better cell survival.

Neuroinflammation is a well-known immune response to protect brain tissue and neurons from toxic molecules or noxious structures in all neurodegenerative diseases and acute brain insults. Still, when overwhelming or prolonged, it may turn harmful and promote neuronal damage and loss^42–45^. An important inflammatory pathway is the metabolism of arachidonic acid (AA), a major component of cell membrane lipids, into precursors of bioactive pro-inflammatory mediators, such as the eicosanoids, prostaglandins, interleukins, TNF and leukotrienes, that promote inflammatory cascades^46,47^. There is a complex relationship between ferroptosis, AA metabolism, and pro-inflammatory mediators. Ferroptotic cell death has been reported to be enhanced by pro-inflammatory polarization of macrophages and microglia^48^. In addition, previous studies indicate that, at least in cancer cells, ferroptosis can have pro-inflammatory effects and initiate inflammation by increasing the expression of PTGS2 (which encodes cyclooxygenase-2 (COX-2)), accelerating AA metabolism and promoting the secretion of pro-inflammatory molecules^49–51^. On the other hand, inflammatory cytokines (such as TNF, PGE2, IL-1, IL-1β, and IL-6) have been shown to directly alter the level and activity of GPx4 in cancer cells^52^ and TNF treatment of cells leads to the sustained downregulation of GPx4 that may trigger ferroptosis^50^. The reduced secretion of MCP-1 and TNF-α, measured from ADA-409-052-stimulated BV2 cells using the BD CBA mouse inflammation kit, further indicates that the compound improves the activity of GPx4, countervailing pro-inflammatory and ferroptotic conditions. To further prove the compound’s efficacy, we tested ADA-409-052 on neuronal death in a neuron (N2a)—macrophage (RAW 264.7) co-culture model of LPS/IFN-γ-induced neuroinflammation. Our data show that ADA-409-052 suppresses pro-inflammatory activation of microglia- and macrophage-mediated neuronal death. Our findings are in line with previous studies showing that intracerebral injection of ferrostatin-1, a ferroptosis inhibitor, reduced the expression of COX-2 with concomitant neuroprotection^20^. This indicates that ADA-409-052 and established ferroptosis inhibitors reduce inflammation and inflammation-induced injury also in brain tissue.

To investigate whether the brain penetrating compound ADA-409-052 can provide neuroprotection in vivo, we chose thromboembolic stroke in mice, as this model results in distinct and relatively fast brain injury and as it mimics the type of stroke that is very common in clinics^53,54^. The model evolves a rather small, focal lesion ensuring a good survival rate and its mechanisms resemble those of vascular occlusion in patients^55^. We also measured the blood flow during and after thrombin injection to secure proper and consistent clot formation, which we found to be reflected as very homogenous infarct volumes measured by MRI analysis, indicating the model’s reliability. We found that treatment with ADA-409-052 resulted in a marked neuroprotection as detected by significantly reduced lesion volumes post-ischemic stroke, which equaled to the reduction obtained by our positive control, minocycline. The protective effect of minocycline replicated in our study the results of previous studies^56–58^. Importantly, only ADA-409-052 restricted the expansion of stroke-induced edema. This effect of ADA-409-052 is in line with the previous finding that intracerebral injection of a ferroptosis inhibitor reduces brain inflammation in a rat model of intracerebral hemorrhage^20^.

Brain swelling is an important clinical measure as it is associated with deterioration and manifestation of neurological deficits and elevated mortality^59,60^. Our data thus confirm the previous findings that ferroptosis is an important mechanism of both brain infarction and brain edema. Furthermore, the results suggest that ADA-409-052, a small molecule inhibiting ferroptotic neuronal death, may be able to efficiently prevent ischemic neuronal death and harmful stroke-induced edema in thromboembolic stroke and prove beneficial in various brain diseases.

Minocycline was used as our reference compound in the stroke study as well as in key in vitro experiments. While we could have compared ADA-409-052 with established inhibitors of ferroptosis, we preferred the compound minocycline, which is extensively characterized and tested in a large number of studies, including our own experiments. It is a small molecule with excellent brain penetration and proven efficacy in vivo^27,58,61–67^. Moreover, minocycline inhibits LP, attenuates iron-induced brain injury following experimental intracerebral hemorrhage^66,68^, and results in expression of molecules counteracting the development of ferroptosis^67^. It is also one of the most potent small molecules against experimental ischemic stroke supported by a large number of studies^27,69,70^. Our data show that minocycline protected against ferroptotic death only at relatively high concentrations, which is in line with previous studies suggesting that it is not a direct ferroptosis inhibitor. Assuming ferroptosis is a major mechanism of stroke-induced secondary edema, it is unsurprising that ADA-409-052 and not minocycline significantly mitigated the brain edema that developed 24 h after ischemic insult in this study’s TE model of stroke.

ADA-409-052 down-regulated pro-inflammatory cytokines and chemokines induced by TE stroke as measured by qPCR, the BD CBA respectively. Among the genes with reduced expression after ADA-409-052 treatment was *Ccl2* (MCP-1), a marker of activated pro-inflammatory microglia/macrophages and infiltrating neutrophils, associated with neuronal degeneration^71^. Even though, ADA-409-052 was unable to change the peripheral secretion of MCP-1, only minocycline caused a certain increase of MCP-1 secretion into the plasma. Importantly, *Il6*, a pro-inflammatory downstream product of toll-like receptor (TLR)^72^, was reduced in both gene expression of ischemic brain tissue and cytokine levels of blood plasma. In addition, ADA-409-052 reduced the expression of stroke-induced *Hmox1* that encodes heme oxygenase-1, an essential enzyme for iron-dependent LP during ferroptotic cell death^73^. These data are in line with in vitro data indicating that ADA-409-052 efficiently inhibits ferroptosis and associated inflammation in brain tissue.

In conclusion, small molecule inhibitors of ferroptosis such as ADA-409-052 may be potent protective compounds against neurodegeneration and acute brain insults. As mechanisms of these brain diseases are complex and involve several detrimental signaling pathways, an advantage of ferroptosis inhibitors is their ability to reduce not only ferroptosis triggered by non-enzymatic LP, but also cell death mediated by enzymatic LP heavily contributed by inflammation. A combination of ferroptosis inhibitors with molecules targeting still other harmful pathways might offer attractive therapeutic approaches for brain disorders.

## Materials and methods

### In vitro lipid peroxidation analysis

C11-BODIPY 581/591 (ThermoFisher Scientific, MA, USA), was used as a fluorescent sensor to determine TBHP-induced LP in PC-12 cells. Analyses were done by cellular imaging or flow cytometry, as C11-BODIPY’s fluorescence emission peak shifts from ~ 590 nm to ~ 510 nm upon oxidation^74^.

For the fluorescent cellular imaging, PC-12 cells (ATCC, VA, USA; for all experiments see suppl. information for cell culturing details) were pre-incubated 90 min with 5 µM C11-BODIPY. Next, cells were washed and exposed for 90 min to 1 mM TBHP (Sigma, MO, USA) in the presence or absence of ADA-409-052 at different concentrations (0.625, 1.25, 2.5, 5, and 10 µM); DMEM media served as control. Images were taken using the 10X magnification at a Zeiss Observer.Z1 fluorescent microscope (AxioCam MRm, Zeiss Zen Imaging Software, Zeiss, Germany). Using the ImageJ software (NIH, MD, USA) an appropriate threshold was set and the mean grey value for each channel (8-bit images) was calculated. Results are displayed as the red/green-ratio, normalized to control.

For the flow cytometry experiments, PC-12 cells were incubated with 500 µM TBHP + /- 10 µM ADA-409-052 for 60 min. Stimulants were replaced with DMEM media containing 5 µM C11-BODIPY for 30 min, before cells were washed with 1X PBS containing 3% iFBS, and resuspended in 1X PBS containing 1% iFBS. Samples were measured on a BD FACSCalibur (488- and 633-nm lasers, standard configuration, BD Bioscience, CA, USA). Data were analyzed using FCS Express v5 (DeNovo Software, CA, USA).

### In vitro ferroptosis models

To test the anti-ferroptotic properties of ADA-409-052, PC-12 cells were exposed for 24 h to either 0.25 µM RSL3 (Selleck Chemicals, TX, USA) or 20 mM glutamate (Glu, Sigma) with or without ADA-409-052 at following concentrations: 2.5, 5, 10, and 20 µM. Minocycline (Sigma) was used in parallel at the same concentrations. The compounds’ efficacy in cell protection was measured as cell viability 24 h post-treatment using the resazurin assay. In brief, cells were incubated for 2 h at 37 °C with 10 µM resazurin in HBSS. The absorbance was measured at 485 nm. Additionally, ADA-409-052’s effect on intracellular GSH levels was measured. We thus exposed PC-12 cells to 20 mM glutamate with or without 5 µM ADA-409-052. After 24 h of exposure, GSH was extracted from the collected cell pellet with 5% sulfosalicylic acid and the supernatant neutralized with 3 M Tris. The Amplite Rapid Fluorimetric Glutathione GSH/GSSG Ratio Assay Kit (AAT Bioquest, Sunnyvale, CA, USA) was used to measure the GSH concentration according manufacturer’s instructions. All results were expressed as percentage of mean fluorescence from untreated control cells.

### Visualization of mitochondria in an in vitro model of ferroptosis

To demonstrate the effect of ADA-409-052 on mitochondria changes upon ferroptosis, PC-12 cells were exposed to glutamate (20 mM) in presence or absence of ADA-409-052 (10 µM) for 24 h, as described before. Live cells were stained with MitoTracker Red CMXRos (ThermoFischer Scientific) and imaged with a 63X Achroplan objective on a Zeiss Laser Scanning Microscope 710 with Zen Imaging Software (Zeiss) (see suppl. information for the full protocol).

### In vitro inflammation model on BV2 microglial cells

BV2 microglial cells were stimulated with pro-inflammatory LPS. Therefore, BV2 cells were incubated with 50 ng/ml LPS (serotype O111:B4, Sigma) for 24 h in the presence or absence of ADA-409-052 using 2.5, 5, 10, and 20 µM. NO release was assessed using the Griess reaction (Promega, WI, USA). A standard curve was prepared using 0–100 μM sodium nitrite. Nitrite concentrations were presented as percentages of NO release after normalization to LPS-exposed BV2 cells. Cytokines were measured from conditioned media of LPS- + /− ADA-409-052-treated cells using the mouse inflammation kit of the BD CBA was performed according manufacturer’s instructions (for details see suppl. information). Data were acquired on a BD Accuri C6 flow cytometer, and the FCAP Array Software v3.0 was used for analyses (all BD Bioscience). Data were normalized to control-treated cells, set as 100%.

### In vitro co-culture model of neuroinflammation using N2a and stimulated RAW 264.7 macrophages

For co-culture experiments, mouse neuroblastoma Neuro-2a (N2a) cells were seeded in a 1:1 ratio with RAW 264.7 macrophages and treated for 24 h with 25 ng/ml LPS and 25 ng/ml IFN-γ (Sigma) with or without ADA-409-052 or minocycline (both 5 µM). Next, cells were collected and incubated with CD11b-Alexa Fluor 647 (1:200, BD Biosciences) for 30 min in the dark at 4 °C, washed with HBSS containing 3% iFBS, resuspended in HBSS with 1% iFBS and counterstained with propidium iodide (PI, 25 µg/ml, Sigma). Samples were analyzed with CytoFLEX S (Beckman Coulter, IN, USA). The surviving/dead N2a cells were detected as Alexa Fluor 647/CD11b negative (CD11b^-^) and PI negative (PI^-^/surviving), and Alexa Fluor 647/CD11b negative (CD11b^-^) and PI positive (PI^+^)/dead, respectively. N2a cell viability was calculated as a fraction of surviving cells to the total number of gated, normalized to control, presented as percentages.

### In vivo experiments

#### Pharmacokinetics of ADA-409-052

Brain penetration study was carried out by TCG Lifesciences Ltd (Kolkata, India) in male BALB/c mice (6–8 weeks old; 18–20 g, in-house breeding). All procedures were in compliance with the guidelines of the Committee for Purpose of Control and Supervision of Experiments on Animals India and the study was approved by the Institutional Ethics Committee of Eurofins Advinus Limited (Approval No.: 019/July-2019). The experiment was carried out in compliance with the ARRIVE guidelines. Mice were housed individually under 12/12 h light–dark cycle with free access to food and water. In two experiments 10 mg/kg or 30 mg/kg of ADA-409-052 (HPLC Purity: 99.7%) dissolved in Tween-80 (0.5%, Merck, Germany) -methylcellulose (Sigma) solution was administered as a single bolus by oral gavage (p.o.). ADA-409-052- or vehicle-administered mice were sacrificed 0.75, 4, and 24 h later (n = 3/group). Heparinized blood samples were stored as plasma at − 80 °C. Whole-brain samples were collected and stored at − 80 °C until homogenization in water (1:4 = tissue: H_2_O, dilution factor: 5), on ice directly prior to analysis. Bio-analysis was performed with API 4000 (AB Sciex Instruments, CA, USA) integrated to LC (Shimadzu, Japan) and CTC PAL (HTS, UT, USA) autosampler and quantified using Analyst 1.4.2 software (AB Sciex).

### Thromboembolic mouse model of ischemic stroke

In accordance with the Council of Europe Legislation and Regulation for Animal Protection, the National Animal Experiment Board of Finland approved the experiments. The experiments were carried out in compliance with the ARRIVE guidelines. Sixty C57BL/6J mice were randomized into four treatment groups using QuickCalcs (GraphPad Software, CA, USA): I: ADA-409-052 (100 mg/kg in 10 ml/kg methylcellulose), II: methylcellulose (10 ml/kg, vehicle for ADA-409-052), III: minocycline (60 mg/kg (1st dose) or 45 mg/kg (2nd dose) in 0.2 ml saline), and IV: 0.9% NaCl (0.2 ml/kg, vehicle for minocycline). ADA-409-052 and methylcellulose were administered every 4 h p.o. for 24 h, starting 60 min before TE surgery. Minocycline and saline were injected intraperitoneal (i.p.) every 12 h (for details see Fig. 5a). All animals underwent TE cerebral ischemia according to Orset et al., 2007^75^. Briefly, 1 µl of 1 IU of α-thrombin (~ 2970 NIH-units/mg) was injected into the MCA-lumen (Fig. 5b). Using Laser Doppler flowmetry, proper clot stabilization was ensured. Blood gases were evaluated in six ischemic mice (for a detailed protocol, see suppl. information).

### Magnetic resonance imaging

Lesion and edema volumes were determined 24 h post-ischemia, using T2-weighted MRI imaging in the anaesthetized animal. MRI was performed on a vertical 9.4 T Oxford NMR 400 magnet as described elsewhere^76^. Multi-slice T2 weighted images (repetition time 3000 ms, echo time 40 ms, matrix 128*256, FOV 19.2 mm, 12 slices à 1 mm-thickness) were obtained. The MRI images were analyzed using an in-house aedes software under MATLAB environment (Math-Works, MA, USA). Lesion and edema volume were calculated blinded to study groups following Shuaib et al.^77^.

Following MRI, various samples were collected from finally anaesthetized animals (details in suppl. information).

### Quantitative real-time PCR analyses of mRNA levels

Total RNA was isolated from homogenized peri-ischemic brain tissue samples of the ipsi- and contralateral hemispheres, in TRIzol (Life technologies, CA, USA) according to the manufacturer’s instructions. cDNA (2.5 ng/µl) was synthesized using 500 ng of total RNA, dNTP, Maxima reverse transcriptase, and random hexamer primers, combined with ribonuclease inhibitors (ThermoFisher Scientific). The relative expression levels of mRNAs encoding the selected genes were measured. According manufacturer’s protocol by RT-qPCR (StepOnePlus, ThermoFisher Scientific) using specific TaqMan gene expression assays (ThermoFisher Scientific), the following genes were analyzed: *Gfap, Arg1, Mpo, Il6, Il10, Tnfα, Ccl2*, and *Hmox*1. The expression levels were normalized to *b2m* (beta-2 microglobulin), calculated as described previously^78^, presented as fold change.

### Analyses of peripheral cytokines in blood plasma

The mouse inflammation kit of the BD CBA (BD Bioscience) was used to detect cytokines secreted into the plasma of 7 mice per group at 1 dpi. CBA was performed according manufacturer’s instructions and data were acquired as described in suppl. information. Results are presented in pg/ml final concentration.

### Statistical analysis

Statistical significant differences (p-values) between the means for two measurement groups were calculated using two-tailed distribution and two-sample equal variance of the student’s t-test; One-way ANOVA with Tukey’s multiple comparison test was used whenever appropriate (GraphPad Prism software). The data are displayed as mean ± standard error of the mean (s.e.m.).

Altogether eight mice (13.34%) were excluded due to mortality, hemorrhagic transformation, or insufficient clot formation.

## Supplementary Information

Supplementary Information.

## Data Availability

The datasets generated and analyzed during the current study are available from the corresponding author on reasonable request.

## Abbreviations

Aif1: Allograft inflammatory factor 1
AD: Alzheimer’s disease
AA: Arachidonic acid
Arg-1: Arginase-1
CCL2: Chemokine (C–C motif) ligand 2
COX-2: Cyclooxygenase-2
DAMPs: Damage-associated molecular patterns
Fe^2^^+^/Fe^3^^+^: Free iron (ferrous/ ferric iron)
GFAP: Glial fibrillary acidic protein
GSH: Glutathione
GPx4: Glutathione peroxidase 4
iFBS: (Heat-) inactivated fetal bovine serum
iNOS: Inducible nitric oxide synthase
IFN-γ: Interferon gamma
ICH: Intracerebral hemorrhage
i.p.: Intraperitoneal
Iba1: Ionized calcium-binding adapter molecule 1
LP: Lipid peroxidation
LPS: Lipopolysaccharide
MFI: Median of the green fluorescence intensity
MCA: Middle cerebral artery
MCP-1 (CCL2): Monocyte chemotactic protein 1
NO: Nitric oxide
p.o.: Oral gavage/per os
PD: Parkinson’s disease
PI: Propidium iodide
PTGS2: Prostaglandin-endoperoxide synthase 2
ROS: Reactive oxygen species
RNS: Reactive nitrogen species
s.e.m.: Standard error of the mean
TE model: Thromboembolic stroke model
TBHP: *tert*-Butyl hydroperoxide
TLR: Toll-like receptor
TNF-α: Tumor necrosis factor alpha
tMCAO: Transient middle cerebral artery occlusion
