# Supplementary material for: An arylthiazyne derivative is a potent inhibitor of lipid peroxidation and ferroptosis providing neuroprotection in vitro and in vivo
Source: Sci Rep. 2021 Feb 10;11:3518. doi: 10.1038/s41598-021-81741-3 (PMC7876050; doi:10.1038/s41598-021-81741-3)
Supplement: Supplementary file 1 — Supplementary Information. [file 41598_2021_81741_MOESM1_ESM.docx]

**An arylthiazyne derivative is a potent inhibitor of lipid peroxidation and ferroptosis providing neuroprotection *in vitro* and *in vivo*.**

Meike Hedwig Keuters^1, 3^ (meike.keuters@helsinki.fi), Velta Keksa-Goldsteine^1^ (velta.keksa-goldsteine@uef.fi), Hiramani Dhungana^1, 3^ (hiramani.dhungana@uef.fi), Mikko T. Huuskonen^1^ (huuskone@usc.edu), [Yuriy Pomeshchik](mailto:Yuriy%20Pomeshchik)^1^ (yuriy.pomeshchik@med.lu.se), Ekaterina Savchenko^1^ (ekaterina.savchenko@med.lu.se), Paula K. Korhonen^1^ (paula.korhonen@uef.fi), Yajuvinder Singh^1^ (yajuvinder.singh@gmail.com), Sara Wojciechowski^1^ (sara.wojciechowski@kct.fi), Šárka Lehtonen^1, 3^ (sarka.lehtonen@uef.fi), Katja M. Kanninen^1^ (katja.kanninen@uef.fi), Tarja Malm^1^ (tarja.malm@uef.fi), Jouni Sirviö^2^ (jouni.sirvio@sauloner.com), Anu Muona^2^ (anu.muona@arandapharma.com), Milla Koistinaho^2^ (milla.koistinaho@helsinki.fi), Gundars Goldsteins^1^ (gundars.goldsteins@uef.fi), Jari Koistinaho^1, 3^ (jari.koistinaho@helsinki.fi)

^1^ A.I. Virtanen Institute for Molecular Sciences, University of Eastern Finland, Kuopio, Finland.

^2^ Aranda Pharma Ltd., Kuopio, Finland.

^3^ Neuroscience Center, University of Helsinki, Helsinki, Finland.

**Supplementary information 1– Material and Methods:**

**Cell culture, plating details:**

***PC-12 cell culture***

PC-12 cells were plated on 96-well plates for 48 h at the density of 1*10^4^ cells/well in DMEM GlutaMAX Gibco, supplemented with 5% heat-inactivated fetal bovine serum (iFBS) and 5% horse serum before cells were assigned to the experimental approach. The density of 1*10^4^ cells/well was kept for all approaches, with the exception of the flow cytometry experiments. Here, PC-12 cells were plated 48 h in advance to the experiment on 6-well plates at a density of 1-2*10^6^ cells/well in full DMEM GlutaMAX Gibco media. All stimulation were done in serum-free DMEM GlutaMAX Gibco media.

***BV2 microglial cell culture***

BV2 cells were plated 24 h on 96-well plates at a density of 9*10^3^ cells/well (for NO-measurement) or on 24-well plates at a density of 6*10^4^ cells/well (for Cytometric Bead Array, CBA) in RPMI‑1640 medium containing 10% iFBS, 4 mM L‑glutamine and 5 µg/ml gentamicin.

**N2a - RAW 264.7 macrophage co-culture**

For co-culture experiments, mouse neuroblastoma Neuro-2a (N2a) cells were seeded in a 1:1 ratio with RAW 264.7 macrophages for 24 h at a density of 2*10^5^ cells/well on 12-well plates in DMEM containing 10% iFBS and 1% penicillin/streptomycin.

**Visualization of mitochondrial changes in an *in vitro* model of ferroptosis**

For the visualization of mitochondria of PC-12 cells during ferroptosis, we plated cells and exposed the cells to 20 mM glutamate in presence or absence of ADA-409-052 (10 µM) for 24 h, as described before. Next, we stained the cells with 10 nM MitoTracker Red CMXRos (ThermoFischer Scientific) for 20 min at 37 $℃$ and 5% CO_2_ in the dark. After one wash, live cells were resuspended in 200 µl media and transferred to a Poly-L-lysine coated 1.3 mm-cover glass. Without delay, the cells were imaged with an Achroplan IR 63x/0.90 W objective on a Zeiss Laser Scanning Microscope 710 with Zen Imaging Software (Zeiss, Oberkochen, Germany). Images were taken as z-stacks (0.5 – 1.5 µm intervals, 18-30 slices/ image, averages 8, frame size 512x512) and aligned as z-stacks. Magnified regions of interest (ROIs) were produced using the zoom function of the Imaris software (Oxford Instruments, Concord, MA, USA).

**Cytokine measurements using the mouse inflammation kit of the BD CBA**

The mouse inflammation kit of the BD Cytometric Beads Array (CBA) contains the following cytokines for measurement: IL-10, IL-6, MCP-1, IFN-γ, TNF-α, IL-12p70.

CBA preparation and performance

Before starting the CBA, standards need to be reconstituted in assay diluent and incubated for 15 min, before a serial dilution is prepared from top standard to 1:1024 dilution, plus negative control (assay diluent only). Next, 10 µl of each capture bead per sample plus standards were mixed in a tube and vortexed vigorously. 10 µl of the capture bead mix was pipetted into each well required of a 96-well plate (round bottom) and 10 µl of each sample was added. Samples were either conditioned media samples from BV2 cells (sample collection is described below) or plasma samples, collected from mice at 1-day post-ischemia; in addition, standards were added, and each sample and standard was mixed by pipetting. Before incubating the plate for 2 h in the dark at room temperature, 10 µl of the PE Detection reagent were added to each sample and standard. Next, 200 µl of wash buffer were added to each well, the plate was spun down for 5 min at 200g and the supernatant is carefully aspirated. Finally, 150 µl wash buffer per well were added and mixed by vigorous pipetting. Data were acquired on a BD Accuri C6 flow cytometer according instructions, and the FCAP Array Software v3.0 was used for analyses (all reagents and instruments BD Bioscience, CA, USA).

Analyses of cytokines in the conditioned media of BV2 cells

BV2 cells, plated as described before, were exposed to LPS (50 ng/ml) in presence or absence of ADA-409-052 at 2.5, 5, and 10 µM concentration; control cells remained in media. After 24 h of exposure, conditioned media was collected in duplicates and stored at -70 $℃$ until analysis.

**Thromboembolic mouse model of ischemic stroke**

In accordance with the Council of Europe Legislation and Regulation for Animal Protection, the National Animal Experiment Board of Finland approved the experiments. C57BL/6J mice (66 male, 4 months old, Jackson Laboratory) were housed under a 12/12 h light/dark cycle, with access to food and water *ad libitum*. Appropriate care was taken to minimize possible suffering of the mice.

Sixty mice were randomized into four treatment groups using QuickCalcs (GraphPad Software, CA, USA): I: ADA-409-052 (100 mg/kg in 10 ml/kg methylcellulose), II: methylcellulose (10 ml/kg, vehicle for ADA-409-052), III: minocycline (60 mg/kg (1^st^ dose) or 45 mg/kg (2^nd^ dose) in 0.2 ml saline), and IV: 0.9% NaCl (0.2 ml/kg, vehicle for minocycline). ADA-409-052 and methylcellulose were administered every 4 h p.o. for 24 h, starting 60 min before TE surgery. Minocycline and saline were injected intraperitoneal (i.p.) every 12 h (for details see Fig. 5a).

All animals underwent TE cerebral ischemia according to Orset et al., 2007 ^72^. Therefore, animals were anesthetized with 5% isoflurane and maintained in a surgical state using 2% isoflurane under a 70%/30% NO_2_/O_2_-atmosphere. A feedback-regulated heating system served to maintain the rectal body temperature at 37 ± 0.5°C. Right before surgery, a glass-micropipette (calibrated at 15 mm/μL; Assistent ref. 555/5; Hoechst, Germany), customized with an electrophysiology puller (P-2000, Shutter Instruments, CA, USA), was pneumatically filled with purified murine α-thrombin (Enzyme Research Laboratories, IN, USA). Through a small cranial window, the micropipette was inserted into the bifurcation of the middle cerebral artery (MCA) before 1 µl of 1 IU of α-thrombin (~2970 NIH-units/mg) was injected into the MCA-lumen by applying positive pneumatic pressure. Keeping the pipette in place for 10 min allowed appropriate thrombus-stabilization (Fig. 5b).

Laser Doppler flowmetry was used to confirm stable MCA occlusion. Using a fiber-optic probe (Moor Instruments, UK), mounted to the intact skull bone in the MCA-territory, the cerebral blood flow was measured before and ≥ 10 min after α-thrombin was injected.

Single-use *i-STAT* test cartridges with the i-STAT system (Abbott, IL, USA) were used to evaluate the blood gas levels in six ischemic mice that were randomly assigned to ADA-409-052 or methylcellulose (vehicle) treatment (n = 3 per group). Immediately after TE surgery, the cartridges were charged with full tail-vein blood and the following parameters were measured: pH, pCO_2_, pO_2_, sO_2_ (%), HCO_3_, TCO_3_, glucose, and BE (B).

**Sample collection form ischemic mice at 24 h after TE-surgery**

After MRI imaging, mice were finally anesthetized and fresh full-blood samples were collected by intracardial puncture into buffered 129 mM sodium citrate tubes (blood : anticoagulant ratio was 1:9). Blood samples were immediately centrifuged at 1500g for 15 min and plasma supernatants were spun down at 13000g for 2 min to ensure proper platelet-removal before plasma samples were snap-frozen and stored at -70 °C until analysis. The mice were then sacrificed by transcardial perfusion using ice-cold, heparinized saline. The following brain samples were collected: cerebellum, infarction and peri-ischemic area, plus corresponding samples from contralateral. All samples were snap-frozen on liquid nitrogen and stored at -70 °C until analysis.
